# Clinical and laboratory outcomes of the solid cancer patients reinfected with SARS-CoV-2

**DOI:** 10.2217/fon-2021-0621

**Published:** 2021-11-26

**Authors:** Oktay Ünsal, Ozan Yazıcı, Nuriye Özdemir, Erdem Çubukçu, Birol Ocak, Aytuğ Üner, Ahmet Özet

**Affiliations:** ^1^Department of Medical Oncology, Gazi University Faculty of Medicine, Ankara 06560, Turkey; ^2^Department of Medical Oncology, Uludag University Faculty of Medicine, Bursa 16059, Turkey

**Keywords:** COVID-19, reinfection, SARS-CoV-2, solid cancer

## Abstract

**Introduction:** The objective of this study was to evaluate the clinical and laboratory outcomes of solid cancer patients who were reinfected with COVID-19. **Methods:** Patients who were tested negative on the Coronavirus disease 2019 (COVID-19) PCR test and those with improved clinical conditions after infection with COVID-19 were enrolled in this study. Patients who received a positive COVID-19 PCR test 28 days after the initial positive PCR test were considered as reinfected. **Results:** A total of 1024 patients with the diagnosis of solid malignancy and COVID-19 PCR positivity were examined. The reinfection rate was 3.1%. Mortality rate of reinfection was 34.3%. The serum ferritin and creatinine values in reinfection were found to be significantly higher than the first infection (respectively; p = 0.015, p = 0.014). **Conclusion:** This study has demonstrated one of the first preliminary clinical results of COVID-19 reinfection in solid cancer patients.

## Lay abstract

Solid cancer patients are at a higher risk than general population in terms of Coronavirus disease 2019 (COVID-19) infectivity and COVID-19-associated death and disease. It is also known that COVID-19 infection has a more severe course in immunocompromised patients. Solid cancer patients may be a vulnerable subgroup of patients to reinfection with COVID-19. The rate of reinfection was 3.1% (n = 32) in our study population of 1024 solid cancer patients who were tested positive on a COVID-19 PCR test. The death rate of the patients with solid cancer was 34.3% (n = 11). In addition, we demonstrated that intensive care follow-up is significantly longer during the reinfection period. It was demonstrated that the time between the last dose of chemotherapy for the patients and the reinfection COVID PCR positivity did not affect the death rate. The COVID-19 pandemic has affected people's daily lives and treatments in many aspects. Owing to the high death rate of reinfection, even if cancer patients have reinfection, our approach is to continue cancer treatment as soon as the patient is cured. Finally, we support the priority vaccination of cancer patients.

Severe acute respiratory syndrome coronavirus 2 (SARS-CoV-2), first described in December 2019, has now infected more than 162 million cases with more than 3.3 million deaths [1]. The fluctuating course and increasing number of the cases continue to affect the whole world [2]. Persistent infection and reinfection are becoming more common. Even though the term reinfection is not definitely established, recurrence of the infection has been increasingly reported. The lack of a well-defined consensus description or criteria for describing true reinfection further complicates the situation. Furthermore, it is still unclear whether innate immunity and primary infection caused by Coronavirus disease 2019 (COVID-19) protect against reinfection.

The COVID-19 pandemic has affected people's daily lives and treatments in many aspects. The treatment of cancer patients who were diagnosed during this outbreak has become much more complicated, considering the possibility of severe complications from COVID-19 and the mortality rate from cancer [3,4]. Solid cancer patients are at a higher risk than the general population in terms of COVID-19 infectivity and COVID-19-associated mortality and morbidity [5]. Patients with hematological malignancies, lung cancer, older age and other comorbitidies including obesity are at increased risk for mortality and morbidity associated with COVID-19 infection [6,7]. Furthermore, Özdemir et al showed that the male gender and receipt of cytotoxic treatment within 4 weeks were significant clinical parameters associated with COVID-19 mortality [8]. It is also known that COVID-19 has a more severe course in immunocompromised patients [9,10].

The studies demonstrated that few months after the first episode of COVID-19 infection, immune responses of host are weakened [11–13]. This may explain the host's susceptibility to a second infection or the reactivation of a previous infection. In the studies, the virus-specific immunoglobulin G (IgG) and IgM were assessed in serum samples taken from individuals infected with COVID-19 to explore the acute antibody response to infection [14,15]. In these studies, it was seen that the antibodies began to rise 5–10 days after the onset of infection. It reaches a peak on the following days and contains a proportion of neutralizing antibodies.

Immunocompromised patients may be more susceptible to COVID-19 reinfection, virus reactivation or recurrent viremia due to impaired immune responses to the virus. Reinfection potential of COVID-19 has been a recently discussed issue in literature. In addition to that, solid cancer patients may be a vulnerable subgroup of patients to reinfection with COVID-19. In this article, we aimed to evaluate the outcomes of solid cancer patients who were reinfected with COVID-19. The secondary aim of the study was to compare laboratory parameters during the first and reinfection period in cancer patients.

## Materials & methods

Patients older than the age of 18 with a diagnosis of solid cancers and admitted to two tertiary medical centers were enrolled in the study. COVID-19 infection was confirmed in all patients with COVID-19 reverse transcription polymerase chain reaction (RT-PCR) on nasopharyngeal and oropharynx swabs.

Patients meeting the following criteria were included in the current study: COVID-19 PCR confirmed acute COVID-19 infection, accompanied by at least one negative COVID-19 PCR test and clinical improvement. In addition to that, at least 28 days after the previous positive COVID-19 PCR result, the patient must have a confirmed COVID-19 PCR positive result (with or without symptoms) again. Patients with delayed PCR positivity were excluded from the study. Therefore, at least 28-day time interval was defined as a threshold for reinfection [14]. All the COVID-19 PCR positive solid malignancy patients admitted to two tertiary cancer centers between June–December 2020 were screened. COVID-19 vaccination were started in January 2021, with priority given to healthcare workers. Therefore, the patients included in the study were not vaccinated. The patients who had full filled the reinfection criteria of COVID-19 were included in the study population.

The inclusion criteria were based on the finding that viral spread was at a minimum in most COVID-19 cases on day 28 after the first acute COVID-19 infection [14,16]. As a result, we assumed that there would be no COVID-19 reinfection within the first 28 days. The treatments of the patients were given in accordance with national guidelines during both COVID-19 infections. The study protocol was approved by the ethics committee of Gazi University Faculty of Medicine (2021-187).

### Statistics

Statistical analyses were performed using the SPSS software version 23. Descriptive analyses were performed using medians for non-normally distributed and ordinal variables. The categorical data are shown in numbers and percentages. Non-parametric tests were conducted to compare these parameters and the ordinal variables. The chi-squared test or Fisher's exact test, where appropriate, was used the compare the proportions in different groups. A p-value of less than 0.05 was considered to show a statistically significant result.

## Results

A total of 1024 patients with a diagnosis of solid malignancy and also with COVID-19 PCR positivity were screened. Thirty-two patients who were admitted to two tertiary medical centers between June 1st and December 31st 2020 and who met the reinfection criteria were enrolled in the study. Median age of the patients was 63.5 (32–93) years with a male to female ratio of 1.46. The clinical and demographic characteristics of 32 patients with COVID-19 reinfection and 992 patients without reinfection are shown in Table 1. There was no difference between the groups according to age, gender, cancer diagnosis and number of comorbidities. The median follow-up period of the patients was 11 (4–115) months from the date of cancer diagnosis. The rate of reinfection was 3.1% (n = 32) in our study population of 1024 solid cancer patients with COVID-19 PCR positivity.

**Table 1.** Comparison of the demographic and clinical characteristics of the patients with and without reinfection.

Parameters	Results		p-value
	n^1^:32	n^2^:992	
Age (median, years)	63.5 (32–93)	61(18–94)	0.078
Gender (n-%) – Female – Male	13 (40.6) 19 (59.4)	484 (48.8) 508 (51.2)	0.472
Diagnosis – Lung cancer – Gastrointestinal tract cancer – Breast cancer – Pancreatic cancer – Genitourinary system cancer – Others	9 (28.1) 7 (21.9) 5 (15.6) 3 (9.4) 3 (9.4) 5 (15.6)	262 (26.4) 225 (22.7) 195 (19.7) 32 (3.2) 129 (13) 149 (15)	0.695
Active treatments prior to first COVID-19 infection (n, %) – Chemotherapy – Hormonal therapy – Targeted therapy – No treatment	22 (68.8) 3 (9.4) 1 (3.1) 6 (18.8)	–^†^ – – –	–
Comorbid disease – 0 – 1 – ≥2	12 (37.5) 9 (28.1) 11 (34.4)	326 (32.9) 411 (41.4) 255 (25.7)	0.288

† This data of all patients are not available.

COVID-19: Coronavirus disease-19; n^1^: Reinfected patients; n^2^: Patients without reinfection.

Nine of the reinfected patients (28.1%) were diagnosed with lung cancer, while 7 cases (21.9%) were diagnosed with gastrointestinal tract cancer, 5 cases (15.6%) with breast cancer, 3 cases (9.4%) with pancreas cancer, 3 cases (9.4%) with genitourinary cancer and 5 cases (15.6%) with other solid malignancies. Twenty-two (68.8%) of the patients were receiving chemotherapy, 1 patient was receiving targeted therapy and 3 patients were receiving hormonal therapy prior to the first COVID-19 infection. Also, 6 patients (18.8%) were not receiving any therapy. While 12 cases (37.5%) did not have any comorbid diseases, 9 cases (28.1%) had one comorbid disease such as chronic obstructive lung disease, diabetes mellitus, coronary artery disease, chronic kidney disease and 11 cases (34.4%) had ≥2 comorbid diseases. Furthermore, 8 patients (25%) were using antiaggregant or anticoagulant therapies.

The median time between the last dose of chemotherapy and the first COVID-19 PCR positivity of the patients was 11 (1–40) days. Twenty-one (65.6%) patients were hospitalized during the first COVID-19 infection, and the median follow-up period for the patients who were hospitalized during the first COVID-19 infection was 14 (3–34) days (Table 2). Two patients (6.2%) were followed up in the intensive care unit. Sepsis developed in 2 patients (6.2%) and pneumothorax in 1 patient (3.1%) as serious complications during the first COVID-19 infection. In the first COVID-19 disease, the median time between positive and negative of the COVID-19 PCR test was 12.5 (6–16) days.

**Table 2.** Follow-up departments of patients receiving treatment in the hospital.

Department	First COVID-19 infection, n (%)	Reinfection, n (%)	p/χ^2^ value
Inpatient clinic	19 (90.5)	6 (37.5)	χ^2^ = 9.338
Intensive care unit	2 (9.5)	10 (62.5)	p = 0.002

The median time between the first COVID-19 infection and reinfection was 46 (30–194) days. After the first COVID-19 infection, 7 of the patients could not continue their cancer treatment. Fifteen (47%) patients were taking chemotherapy, 1 (3.1%) patient was taking hormonal therapy and 1 (3.1%) patient was taking targeted therapy in the period between the the first COVID-19 infection and reinfection. Sixteen patients (50%) were hospitalized during the reinfection and the median time of the hospital stay was 21 (3–75) days (Table 2). During the reinfection, 10 patients (31%) were followed up in the intensive care unit. Patients receiving their treatment in the hospital were compared. The number of patients hospitalized in the service in the first COVID-19 infection and followed up in the intensive care unit for reinfection was statistically significantly higher (p = 0.002; Table 2). Sepsis developed in 9 patients (28.1%) and pneumothorax in 1 patient (3.1%), acute coronary syndrome in 2 patients (6.2%) and disseminated intravascular coagulation (DIC) in 1 patient (3.1%) as serious complications during the reinfection. Eleven patients died during the follow-up period because of the complications such as sepsis, DIC and pneumothorax. Mortality rate of reinfection in patients with solid cancer was 34.3% (n = 11). Nine of the patients who died during reinfection were males, and 2 were females. The most common diagnosis of patients who died during reinfection was lung cancer (n = 5). All these 5 patients were males.

The median procalcitonin levels of the patients measured during the reinfection were higher than measured during the first COVID-19 infection, which were statistically not significant (p = 0.07; Table 3). The median ferritin values in the first COVID-19 infection and reinfection were 243.5 (10–4499) and 512.5 (65–2800), respectively (Table 3). Furthermore, median ferritin levels of the patients measured during reinfection were significantly higher than measured during the first COVID-19 infection (p = 0.015; Table 3). The median creatinine values of patients with the first COVID-19 infection and re-infection were 0.8 (0.39–3.45) and 0.94 (0.28–2.33), respectively (Table 3). The absolute difference in creatinine level between those with and without COVID-19 reinfection was 0.14 mg/dl. In addition, median serum creatinine levels of the patients were significantly higher which was measured during reinfection (p = 0.014). Despite the difference of 0.14, it was determined that the reason for the significant difference was mean rank. It was determined that mean rank of creatinine values were significantly higher in patients with re-infection compared to those with the first infections. When the parameters with a significant difference according to the first and reinfection status were examined, the effect size of the ferritin levels in the first and reinfection was 0.088, and the effect size of creatinine levels was determined to be 0.276. Values for other laboratory parameters are shown in Table 3.

**Table 3.** Comparison of the laboratory parameters of the first COVID-19 infection and reinfection.

Parameters	Results (median/min–max)		p-value
	First COVID-19 infection	Reinfection	
CRP (mg/l)	74.6 (5–463)	71.1 (4–301)	0.945
D-dimer (μg/ml)	1.4 (0.32–11.5)	1.88 (0.26–16.23)	0.118
Procalcitonin (ng/ml)	0.3 (0.02–2.06)	0.4 (0.03–8.97)	0.070
Fibrinogen (mg/dl)	413.5 (244–713)	410 (90–785)	0.510
Ferritin (ng/ml)	243.5 (10–4499)	512.5 (65–2800)	**0.015**
Interleukin-6	87.15 (4–204)	64.5 (19–105)	0.655
Leucocyte (×10^3^/μl)	7.48 (3.1–27.33)	8.62 (2.15–51.74)	0.207
Neutrophil (×10^3^/μl)	5.74 (1.32–25.86)	6.29 (1.31–48.9)	0.449
Lymphocyte (×10^3^/μl)	0.83 (0.31–3.02)	0.77 (0.29–5.18)	0.861
Hemoglobin (g/dl)	10.3 (7.1–16.6)	10.35 (7–16.8)	0.581
Platelet (×10^3^/μl)	228.5 (61–873)	229 (35–716)	0.548
LDH (U/L)	275.5 (95–937)	290 (143–1770)	0.340
Creatinine (mg/dl)	0.8 (0.39–3.45)	0.94 (0.28–2.33)	**0.014**
Albumin (g/dl)	3 (1.5–4.9)	3.05 (2–5.1)	0.637

p-values of less than 0.05 are in bold.

Eight patients were taking anticoagulant or antiaggregant therapy for a long time. While 2 of the patients (25%) who were using chronic antiaggregant or anticoagulant agents died, 9 of the patients (37.5%) who were not using any antiaggregant or anticoagulant agents died during the follow up which was not statistically significant (p = 0.681). Nine patients with only 1 comorbidity had higher mortality (p = 0.052; Table 4). While 36.4% of patients (4/11) who received the last chemotherapy dose within one month ago prior the reinfection died, 35.7% of the patients (5/14) who received the last chemotherapy dose more than 3 months before reinfection died. There was no significant association between the time of the last chemotherapy dose and mortality during reinfection (p = 0.935).

**Table 4.** Statistical analysis of mortality and some clinical parameters.

Parameters	Died (n = 11) n (%)	Alive (n = 21) n (%)	p/χ^2^ value
Diagnosis – Lung cancer – Others	5 (45.5) 6 (54.5)	4 (19) 17 (81)	p = 0.213
Comorbid disease – No – 1 – ≥2	3 (27.3) 6 (54.5) 2 (18.2)	9 (42.9) 3 (14.2) 9 (42.9)	χ^2^ = 5.906 p = 0.052
Chronic anticoagulant or antiaggregant use – Yes – No	2 (18.2) 9 (81.8)	6 (28.6) 15 (71.4)	p = 0.681
Time from last chemotherapy to reinfection positivity – <1 month – 1–3 months – >3 months	4 (36.4) 2 (18.2) 5 (45.4)	7 (33.3) 5 (23.8) 9 (42.9)	χ^2^ = 0.135 p = 0.935

## Discussion

In the current study, we demonstrated that patients with comorbidities have statistically higher mortality after reinfection. In addition, we observed that ferritin and creatinine values were significantly higher in re-infection with COVID-19 in solid cancer patients. In patients with solid tumors, the rate of COVID-19 reinfection was 3.1%. The mortality rate of reinfection in patients with solid cancer was 34.3%. Also, we demonstrated that intensive care follow-up period was significantly longer during the reinfection period. This study demonstrated one of the first preliminary clinical results of COVID-19 reinfection in solid cancer patients. Previously, there have been cases reported previously in patients with a hematological malignancy [17,18].

In the current study, the reinfection rate was 3.1% in solid cancer patients. In the analysis by Piri *et al*., the recurrence rate of COVID-19 infection in the normal population ranged from 2.3% to 21.4% [19]. In this systemic review, the median time until reinfection of the patients was 20 days (1–98). In our study, the median period was 46 days (30–194). This difference might be associated with the inclusion criteria of the current study.

The most common cancer subtype among the patients with reinfection was lung cancer in this study. A recent study reported that the first COVID-19 infection was more common in patients with breast cancer. While 19.8% of cancer patients with reinfection had a diagnosis of breast cancer in that study [8], in our study, the percentage of patients with breast cancer with reinfection was 15.6%. It was also found that the patients with lung cancer were at a higher risk of mortality during reinfection.

According to the data of the WHO dated April 25, 2021, the mortality rate due to COVID-19 infection is currently 2.1% [1]. This continues to decrease in comparison to previously reported rates. Different results have been reported regarding the mortality rates of cancer patients in COVID-19 infection. It has also been reported that cancer patients have a higher mortality compared to other patients. The first study on this topic was published in China, and the mortality rate was reported as 28.6% in cancer patients [20]. The mortality rate was 20% in two other following studies [21,22]. The COVID-19 and Cancer Consortium (CCC19) study reported a 12% mortality rate in solid tumors and 14% in hematological malignancies [23]. The meta-analysis of Giannakoulis *et al*. showed an increased mortality in patients with cancer compared to those without (13.5% vs 5.1%) [24]. In the current study, the mortality rate due to reinfection was 34.3%. According to the mortality rates in all studies, the mortality rate of reinfection was higher compared to first COVID infection attack in solid cancer patients. It is difficult to create a general statement due to the small number of patients in the current study. Furthermore, the rate of mortality might be affected by the differences in treatment algorithms between the medical centers and by comorbitidies of the patients. In the study, the mortality of cancer patients with one additional comorbid disease was significantly higher than the others. Four of these patients had chronic obstructive pulmonary disease (COPD). These were the patients using chronic oxygen therapy prior to the reinfection.

More patients were hospitalized during the first COVID-19 infection than the reinfection. However, these patients had better prognosis. As the indications for hospitalization changed over time according to national guidelines, fewer patients received inpatient treatment for reinfection, but they had a more severe course. The hospital stay and intensive care unit follow-up were longer due to the reinfection. The length of hospital stay of the patients at the time of first and reinfection was not compared. Because in both COVID-19 infections, the number of inpatients in the hospital was 13. Since this number is small, statistical analysis was not made. The rate of patients followed up in the intensive care unit due to reinfection was 31.2% among all patients. This rate was higher than in any other studies due to the first COVID-19 infection [20,21,23–26]. Zhang *et al*. reported the rate of intensive care hospitalization as 21.6% in cancer patients due to COVID-19 infection [20]. In another study by Yang *et al*., this rate was 20% [21]. This data can be interpreted as reinfection is more severe disease course which may need more frequent intensive care unit stay. Reinfection might have heavy load on healthcare systems by increasing the intensive care bed occupancy rate. Therefore, to prevent reinfection in this vulnerable subgroup of the patients, intensive and urgent vaccination strategies might be organized in solid cancer patients.

In addition, it was detected that mortality would be higher in cancer patients who recently received chemotherapy in our study. Many studies have conflicting results on this issue. CCC19, TERAVOLT and UKCCMP studies did not show any association between recent cytotoxic therapy and mortality, while Yang *et al.* and Özdemir *et al.* showed a positive association [8,21,23,25,27]. However, it was demonstrated that the time between the last dose of chemotherapy of the patients and the re-COVID PCR positivity did not affect the mortality. Most of the patients who received cytotoxic chemotherapy continued their treatment after the first COVID-19 infection to prevent the serious cancer-related morbidity and mortality. Therefore, even if cancer patients have reinfection, our approach is to continue cancer treatment as soon as the patient is cured. Targeted therapy and hormone therapy were also continued in the patients with reinfection in this study. As the number of patients using medications in these two therapy groups was small, we could not analyze this different therapy groups. It was difficult to make further interpretation about the continuation of these therapeutics.

Laboratory parameters in cancer and the COVID-19 infection were evaluated in different studies. It has been suggested that COVID-19 infection is characterized by an exaggerated and dysfunctional immune response “cytokine storm” that results in multiple organ damage and death. One study also showed that patients with acute respiratory distress syndrome (ARDS) due to COVID-19 infection had lower blood levels of proinflammatory cytokines compared to patients with ARDS from other causes [28]. As cancer patients may have baseline abnormalities in blood counts and inflammatory markers, this may have different effects compared to those observed in non-cancerous COVID-19 patients. Higher levels of inflammatory markers, neutrophil lymphocyte ratio (NLR), pro-calcitonin levels, ferritin, CRP and lower levels of albumin (as a negative acute phase reactant) have shown to be associated with higher mortality [20,22,29]. Many of these markers are known to be poor prognostic factors for cancer patients [30]. Median levels of serum ferritin was found to be significantly higher during reinfection compared to the first infection in this study. The procalcitonin and D-dimer levels were higher during reinfection than the first infection, which was not statistically significant. The study conducted by Terpos *et al.* in patients with COVID-19, they showed that procalcitonin is a poor prognostic factor [31]. Moreover, levels of serum creatinine were significantly higher in reinfection. As a result of the analysis, it was found that the effect value of creatinine levels was higher. An analysis of prognostic factors in patients infected with COVID-19 showed that increased creatinine levels were associated with poor prognosis [32]. Similar to our study, studies on the ferritin levels have shown that it is a poor prognostic for the COVID-19 infection [31,32]. The increased ferritin and creatinine values in this study may have increased as a result of the course of the COVID-19 re-infection. Therefore, ferritin and creatinine values in reinfected patients should be interpreted with caution. This study found that an increase in ferritin and creatinine values may be the result of COVID-19 reinfection, not the cause.

Association between the mortality and chronic anticoagulant or antiaggregant usage in the patients during reinfection was not significant due to the small number of the patients using chronic anticoagulant or antiaggregant agents. The COVID-19 infection may predispose to venous or arterial thromboembolism due to increased inflammatory response, hypoxia, immobilization, and the presence of DIC [33–35]. It suggests that the coagulopathy associated with COVID-19 is a combination of low-grade DIC and pulmonary thrombotic microangiopathy that can have a serious impact on organ dysfunction in the most patients with severe disease [36]. Rate of patients with DIC and septic shock of reinfected patients were higher compared to the first COVID-19 infection. Considering that cancer patients are more prone to thrombosis, chronic anticoagulant or antiaggregant use may be considered protective. However, we could not provide a meaningful result due to our small sample size.

This study had some limitations. First, we could not prove that the virus is re-transmitted from another strain, as we did not sequence the virus in every segment. Second, we could not quantify the neutralizing antibodies in the patients. In addition to that, we had only limited number of reinfected patients.

## Conclusion

The current study demonstrated that COVID-19 reinfection mortality was 34.3%, and reinfection rate was 3.1% in patients with solid cancer. The serum ferritin and creatinine levels were increased during reinfection compared to first attack. In addition, during the reinfection, the number of patients followed in the intensive care unit and the intensive care follow-up periods were higher. Therefore, to prevent reinfection in this vulnerable subgroup of patients, intensive and urgent vaccination strategies might be organized in solid cancer patients. We think that the preliminary results of the current study would open new horizons to define the possible risk factors for reinfection with COVID-19 in patients with solid cancer.

**Summary points**

- The rate of reinfection was 3.1% (n = 32) in our study population of 1024 solid cancer patients with COVID-19 PCR positivity.
- The median time between the first COVID-19 infection and reinfection was 46 (30–194) days.
- Mortality rate of reinfection in patients with solid cancer was 34.3% (n = 11).
- The median serum ferritin levels of the patients measured during reinfection were significantly higher than measured during the first COVID-19 infection (p = 0.015).
- The median serum creatinine levels of the patients were significantly higher than measured during the reinfection (p = 0.014).
- The number of the patients hospitalized in the service in the first COVID-19 infection and followed up in the intensive care unit for reinfection was statistically significantly higher (p = 0.002).
- Nine patients with only 1 comorbidity had higher mortality (p = 0.052).
- There was no significant association between the time of the last chemotherapy dose and mortality during reinfection (p = 0.935).
- While 2 (25%) of 8 patients using chronic antiaggregant or anticoagulant agents died, 9 (37.5%) patients who did not use any antiaggregant or anticoagulant agent died during follow-up, and it was not statistically significant (p = 0.681).
